# Role of Gamma Knife® Radiosurgery for the Treatment of Brain Metastases from Gynecological Cancers

**DOI:** 10.7759/cureus.947

**Published:** 2016-12-31

**Authors:** Andrew Keller, Rahim Ismail, Peter S Potrebko, Julie Pepe, Meiling Wu, Kunal Saigal, Matthew Biagioli, Ravi Shridhar, Robert Holloway, Melvin Field, Nikhil G Rao

**Affiliations:** 1 College of Medicine, University of Central Florida; 2 Radiation Oncology, Florida Hospital; 3 Research Services, Florida Hospital; 4 Quality Improvement, Florida Hospital; 5 Gynecologic Oncology, Florida Hospital; 6 Neurosurgery, Florida Hospital

**Keywords:** brain metastases, stereotactic radiosurgery, gamma knife radiosurgery, gynecological cancers

## Abstract

Objective: Gamma Knife^®^ (GK) (Elekta Instruments, Stockholm, Sweden) radiosurgery is well established for treatment of brain metastases. There are limited data on patients treated with GK from gynecological cancers. The authors sought to determine the effectiveness of the GK in patients with brain metastases from gynecological cancers.

Methods: An IRB-approved database was queried for patients with gynecologic cancers treated with GK between June 1996 and May 2016. Imaging studies were reviewed post-SRS (stereotactic radiosurgery) to evaluate local control (LC) and distant brain control (DC). Overall survival (OS), local control, and distant brain control were calculated using the Kaplan-Meier (KM) method and log-rank test.

Results: Thirty-three patients underwent SRS for 73 separate cranial lesions. The median age was ­58.5 years, and 17 (52%) also had extracranial metastases. Ten (30%) patients had previously received whole brain radiotherapy (WBRT), and 11 (33%) underwent concurrent WBRT. The median tumor volume was 0.96 cm^3^. Median radiographic follow-up was 11 months. At the time of treatment, 39% of patients were categorized as recursive partitioning analysis (RPA) Class I, 55% as RPA Class II, and 6% as RPA Class III. The local failure rate was 8%. Five patients (15%) developed new brain lesions outside the radiation field with a median progression-free survival (PFS) of seven (range: 3-9) months. Median OS was 15 months from GK treatment. One-year OS was 72.9% from GK treatment. Primary cancer histology was a significant predictor of OS, favoring ovarian and endometrial cancer (p = 0.03).

Conclusions: Gamma Knife stereotactic radiosurgery for gynecologic brain metastases leads to excellent control rates of treated lesions. Primary histology may have a significant impact on OS following GK, with improved survival seen with ovarian and cervical cancer following Gamma Knife radiosurgery (p = 0.03).

## Introduction

In the United States, 105,890 cases of gynecologic malignancies will be diagnosed, and 30,890 patients will die in 2016 [1]. Gynecological cancers are an infrequent (< 1%) cause of brain metastases and less than 3% of patients with gynecologic malignancies are diagnosed with central nervous system metastases [2]. Unfortunately, patients who develop brain metastases have a poor prognosis overall and require central nervous system control to improve their quality of life and ability to function [4]. Given the relative inability of systemic therapy to penetrate the blood-brain barrier, radiation therapy and surgical resection play vital roles in the management of brain metastases [3-4]. 

Patients with gynecologic malignancies who develop brain metastases have a poor prognosis [4]. Aggressive treatment with surgery, focal therapy, and systemic therapy may improve patient outcomes [5-8]. Stereotactic radiosurgery with the Gamma Knife® (Elekta AB, Stockholm, Sweden) offers a minimally invasive way to ablate cranial metastases. However, due to the rarity of this presentation, there are only a handful of studies describing outcomes of patients with gynecologic malignancies treated with Gamma Knife [7, 9-11]. Herein, we report the results of the Gamma Knife in the multimodality treatment of patients with gynecological cancers diagnosed with brain metastases.

## Materials and methods

### Patients

This research study was approved by the Institutional Review Board (IRB) of Florida Hospital, approval #907433-1. We performed a retrospective analysis of an IRB approved database of patients treated with Gamma Knife radiosurgery between June 1996 and May 2016 at our institution. Female patients who were specifically treated for brain metastases and who had an established diagnosis of gynecological malignancy were selected for study inclusion. Patient characteristics collected included Karnofsky performance status, extracranial disease status, tumor histology, history of whole brain radiation therapy, history of craniotomy, date of death or last clinical contact, and age at initial stereotactic radiosurgery (SRS) treatment. Treatment characteristics were obtained from the treatment plans, including tumor volume, matrix volume, prescription dose, prescription isodose, coverage, selectivity, and gradient index.

### Gamma Knife stereotactic radiosurgery technique

The brain tumor was visualized on the planning MRI with gadolinium or CT with contrast, and treatment was planned through the use of GammaPlan (Elekta AB, Stockholm, Sweden). Dose selection was based on published Radiation Therapy Oncology Group (RTOG) guidelines, with possible dose modification based on treating physician preference, tumor location, and the number of lesions [12]. All visible metastatic brain disease was treated during each Gamma Knife session. Patients were treated with the Leksell Gamma Knife model U from 1996-2005, the Leksell Gamma Knife model 4C from 2005-2013, and the Leksell Gamma Knife Perfexion™ (Elekta AB, Stockholm, Sweden) from 2013-2016. Head frame placement was done by the treating neurosurgeon with subsequent removal of the frame by either the treating radiation oncologist or neurosurgeon.

### Chart review and follow-up

Patients were typically followed with serial imaging of the brain following treatment every three to six months during the first year, followed by every six months in subsequent years to assess for tumor progression. The date of each SRS session, location of the brain metastasis treated, and greatest axial dimension on the planning MRI or CT at the time of treatment were recorded. Post-radiosurgery imaging was reviewed to identify all brain metastases and to measure the size of the lesion as the greatest axial diameter. Out of the 73 treated brain metastases, 50 had at least one follow-up study available within our institution’s medical records. Thus, the other 23 metastases without follow-up imaging were excluded from brain control analysis.

Actuarial patient survival was defined as the time in months from initial Gamma Knife treatment to date of death or date of last clinical contact (up to June 2016). Distant brain failure was defined as the time in months from the initial SRS to first subsequent radiographic evidence of a new brain metastasis. Local failure was defined as the time at which a brain metastasis greater than 1.20 times its original size was evident and maintained this increase in size. This definition to assess local failure was based on the Response Assessment in Neuro-Oncology Brain Metastases (RANO-BM) criteria, which defines progressive disease as a 20% increase in size [13].

### Statistical analysis

Statistical analyses were performed using the Statistical Package for Social Sciences (SPSS), version 23 (IBM SPSS Statistics, Armonk, NY). Descriptive statistics were used to summarize the cohort. The overall survival, local brain control, and distant brain control rates were calculated from the date of the first Gamma Knife treatment session to the date of death or progression via the Kaplan-Meier (KM) method. Univariate analysis was performed using the log-rank test to assess outcome measures. Due to the sample size limitations, a multivariate analysis was not performed. A two-tailed p-value ≤ 0.05 was considered to be statistically significant. No adjustments were made for multiple tests.

## Results

### Patient characteristics and treatment parameters

We identified 73 metastatic brain lesions treated in 33 patients. Sixty-eight metastases (93%) were intact and five (7%) were postoperative cavities. The clinical characteristics of the 33 patients are outlined in Table 1. The median patient age was 58.5 years (range: 32-93). All patients had been diagnosed with a gynecological malignancy by histology. Seventeen patients (52%) had extracranial metastases. Eleven patients (33%) had progressive systemic disease. All patients underwent Gamma Knife-based SRS. Ten patients (30%) received previous whole brain radiotherapy (greater than three months prior to SRS) and 11 patients (33%) received concurrent whole brain radiotherapy (within three months of SRS session). The median radiation dose to each tumor was 20 Gy (range: 14 - 24 Gy) (Table 2) treated to the 50% isodose line. The median tumor volume was 0.96 cm3 (range: 0.01 – 16.97 cm3). At the time of initial treatment, 39% of patients were categorized as RPA Class I, 55% as RPA Class II, and 6% as RPA Class III.

**Table 1:** Summary of Characteristics in 33 Patients with Gynecological Brain Metastases Abbreviations: WBRT = whole brain radiotherapy; KPS = Karnofsky Performance Status

Variable	No. (%)
No. of patients	33
No. of lesions	73
Radiographic Follow-Up (mos)	
Median	11.4
Range	1–28
Age (yrs)	
Median	58.5
Range	32–93
Primary Disease	
Ovarian Cancer	17 (52)
Endometrial Cancer	10 (30)
Cervical Cancer	6 (18)
KPS %	
90	15 (45.5)
80	15 (45.5)
70	1 (3)
60	2 (6)
RPA Class	
I	13 (39)
II	18 (55)
III	2 (6)
Extracranial Metastases	
Present	17 (52)
Absent	16 (48)
Controlled Primary	
Yes	31 (94)
No	2 (6)
Systemic Disease Status	
Progressive	11 (33)
Stable	5 (15)
Complete Response	17 (52)
Previous WBRT	
Yes	10 (30)
No	23 (70)
Concurrent WBRT	
Yes	11 (33)
No	22 (67)

**Table 2:** Summary of Characteristics in 73 Gynecological Brain Metastases

Variable	No. (%)
Tumor Volume (cm^3^)	
Median	0.96
Range	0.01 - 16.97
Diameter (mm)	
Median	11.8
Range	1.7 - 42.5
Coverage	
Median	1
Range	0.71 - 1.0
Selectivity	
Median	0.61
Range	0.03 - 0.86
Gradient Index	
Median	2.85
Range	0.79 - 4.24
Dose (Gy)	
14	1 (1)
15	2 (3)
16	9 (12)
18	14 (19)
20	11 (15)
24	36 (50)
Local Failure	
Yes	6 (8)
No	44 (60)
No Available Follow-Up	23 (32)
Time to Local Failure (mos)	
Median	8.3
Range	4.0 - 17.4
Location of metastases	
Brainstem	5 (7)
Cerebellum	20 (28)
Frontal	17 (23)
Internal Capsule	1 (1)
Occipital	7 (10)
Paraventricular	1 (1)
Parietal	13 (18)
Parieto-occipital	1 (1)
Temporal	7 (10)

### Local and distant brain control

**Figure 1:**
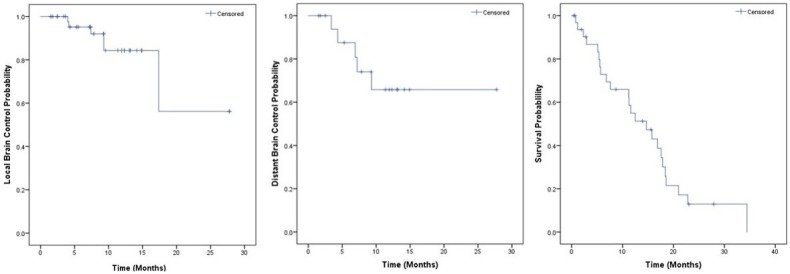
Kaplan-Meier Curves for Local Control, Distant Brain Control, and Survival

**Figure 2:**
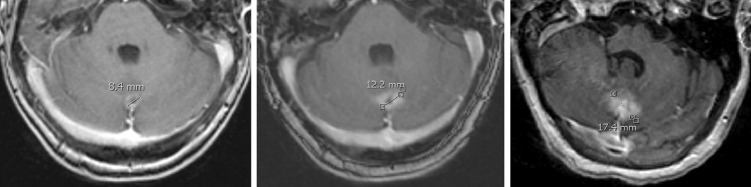
Pre-treatment MRIs (1st image) and Post-treatment MRIs (at 9 months and 15 Months Following GK) of Metastasis Receiving 24 Gy, with Significant Increase in Lesion Size Seen Post-treatment, Consistent with Radiation Necrosis

The median radiographic follow-up was 11 months (range: 1–28 months). The rate of local brain failure as defined was 8%. Kaplan-Meier local control (LC) estimates at six and 12 months were 95.1% and 84.3%, respectively (Figure 1). Results of univariate analysis (UVA) of LC and distant brain control (DBC) are displayed in Table 3. A dose of greater than 20 Gy was found to statistically correlate with the likelihood of increased lesion volume on serial imaging (p = 0.04). Figure 2 provides an example of increased lesion size on imaging seen in a metastasis receiving > 20 Gy, which appears radiographically consistent with radiation necrosis. No other factors appeared to have a statistically significant effect on local control on UVA. No factors appeared to have a statistically significant effect on DBC on UVA.

**Table 3:** Univariate Analysis (UVA) for Local Control, Distant Brain Control, and Survival Abbreviations: WBRT = whole brain radiotherapy; SRS = stereotactic radiosurgery; KPS = Karnofsky Performance Status

	p Value
Local Control	
Histology (Ovarian vs. Endometrial vs. Cervical)	0.8
Pre-SRS WBRT (yes or no)	0.21
Concurrent WBRT (yes or no)	0.37
Postop	0.84
Dose (≤ 20 Gy vs > 20 Gy)	0.04
Tumor Volume (< 4 cc vs ≥ 4 cc)	0.8
Tumor Location (Infratentorial vs Supratentorial)	0.08
Max Diameter Category (< 7.5 mm vs ≥ 7.5 mm)	0.86
Distant Brain Control	
Histology (Ovarian vs. Endometrial vs. Cervical)	0.59
Pre-SRS WBRT (yes or no)	0.87
Concurrent WBRT (yes or no)	0.46
Extracranial Disease Status (Progressive vs Stable vs Complete Response)	0.73
Extracranial Metastases (yes or no)	0.77
Controlled Primary (yes or no)	0.51
Age (< 60 vs ≥ 60)	0.92
Volume of Largest Brain Metastasis (< 4 cc vs ≥ 4 cc)	0.32
Initial Number of Brain Metastases (1 vs > 1)	0.93
Survival	
Histology (Ovarian vs. Endometrial vs. Cervical)	0.03
Pre-SRS WBRT (yes or no)	0.54
Concurrent WBRT (yes or no)	0.74
Extracranial Disease Status (Progressive vs Stable vs Complete Response)	0.14
Extracranial Metastases (yes or no)	0.66
Controlled Primary (yes or no)	0.34
Age (< 60 vs ≥ 60)	0.78
KPS (< 70 vs > 70)	0.07
RPA (Class I vs Class II vs Class III)	0.17
Volume of Largest Brain Metastasis (< 4 cc vs ≥ 4 cc)	0.27
Initial Number of Brain Metastases (1 vs > 1)	0.7

Five patients (15%) were noted to have failure in the brain at a site remote to the GK, with a median time to failure of seven months (range: 3–9 months). Three patients (8%) with distant failures received additional Gamma Knife-based SRS. Kaplan-Meier estimates for DBC at six and 12 months were 87.5% and 65.8%, respectively (Figure 1). 

### Overall survival

**Figure 3:**
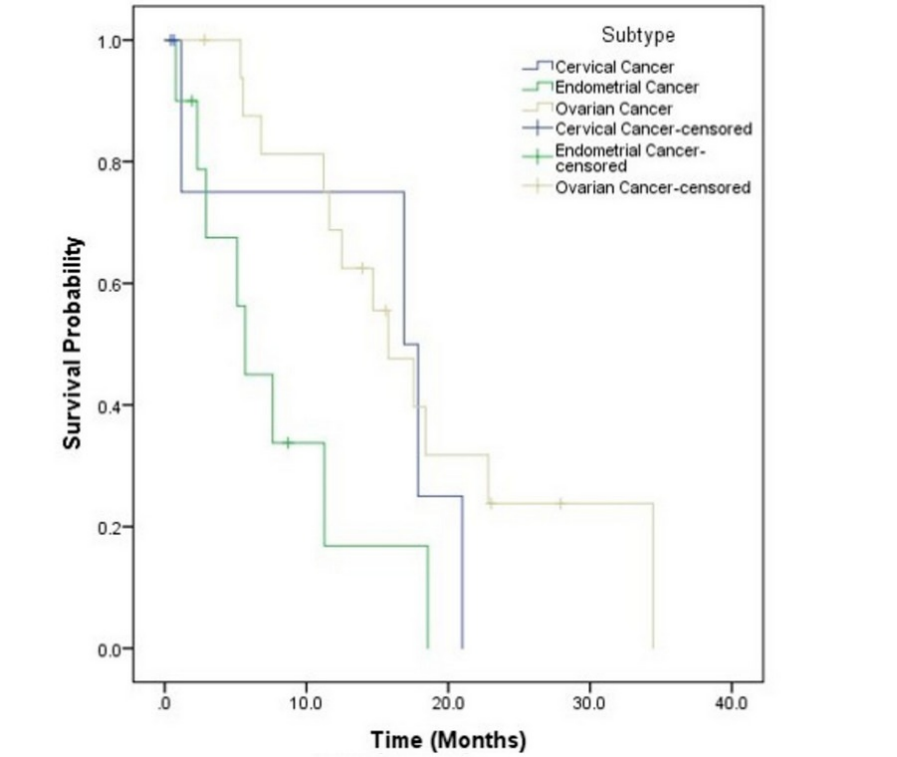
Survival Based on Subtype

The median OS estimate by the KM method was 21 and 15 months from the date of brain metastases diagnosis and the date of SRS, respectively. The OS estimates at six and 12 months from the time of initial SRS treatment were 72.9% and 54.9%, respectively (Figure 1). Primary cancer histology was a significant predictor of KM OS estimates from the date of treatment (p = 0.04), with cervical cancer patients having a median survival of 17 months, endometrial cancer patients having a median survival of six months, and ovarian cancer patients having a median survival of 16 months (Figure 3). No other factors appeared to have a statistically significant impact on OS on UVA. Univariate analyses for OS are shown in Table 3. Although RPA Class did not have a statistically significant impact on overall survival (p = 0.14), median survival was 18 months for RPA Class I, 12 months for RPA Class II, and 2 months for RPA Class III.     

### Toxicity

Radiation necrosis was confirmed via biopsy following surgical resection in two patients (6%). There were no other observed cases of RTOG Grade 3 or higher toxicity following treatment. 

## Discussion

Gynecologic malignancies have a low rate of metastatic dissemination to the brain [2]. Brain metastases that develop typically require a combination of radiation therapy and neurosurgical intervention for control [3-4], and chemotherapy may be helpful in selected cases. Due to the neurocognitive deficits that can result following whole brain radiotherapy, Gamma Knife radiosurgery is often employed for patients with brain metastases, especially those with limited brain disease [3, 14]. Gamma Knife radiosurgery has been shown to be very effective in treating brain metastases from other primary cancers [15-18]. However, there are limited data on patients with brain metastases from gynecological malignancies, given their relatively rare incidence [7, 9-11, 19-20]. Our results demonstrate excellent local control rates without significant acute or late toxicity when Gamma Knife radiosurgery is employed in patients with brain metastases from gynecological malignancies.

The primary outcome of this study was the ability of Gamma Knife radiosurgery to control brain metastases from gynecological malignancies. The excellent local control rates illustrated within our study were consistent with prior reports in the literature [9, 11, 19-20]. Our six and 12-month KM local control estimates were 95.1% and 84.3%, respectively. Our overall local control failure rate was 8%. Matsunaga, et al. reported similar local control rates of 96.4% at six months and 89.9% at 12 months when they used Gamma Knife radiosurgery to treat brain metastases from gynecological malignancies [9]. Thus, we confirm their findings and agree with a potential role for Gamma Knife radiosurgery to treat brain metastases from gynecological malignancies.

A significant predictor of local control was the prescription dose. We noted that a dose of more than 20 Gy was found to statistically correlate with the likelihood of increased lesion volume on serial imaging (p = 0.04), likely due to radiation necrosis. Two of the patients who received doses of 24 Gy and experienced progression based on our criteria had pathologically proven radiation necrosis. The remaining incidences of progression were not biopsied but radiographically were consistent with necrosis (Figure 2).    

Although our local control rates were excellent, our rates of distant brain control were much lower, with KM estimates of distant brain control of 87.5% at six months and 65.8% at 12 months. Surprisingly, prior whole brain radiotherapy and combined whole brain radiotherapy did not appear to have a statistically significant impact on rates of distant brain control in our cohort. Of the five patients who developed distant brain metastatic disease, three were treated with subsequent Gamma Knife radiosurgery. The other two patients who developed distant brain metastatic disease received no additional radiotherapy, having already received prior WBRT and dying shortly after their distant failure was observed radiographically. Our results indicate that it may be safe to omit upfront WBRT for the selected patient with limited volume brain disease. 

Our KM estimates of median overall survival were 21 and 15 months from the date of brain metastases diagnosis and the date of SRS, respectively, consistent with similar series, as shown in Table 4. Additionally, our KM OS estimates were similar to other studies that reported overall survival between 34.7% and 73.9% at six months and between 13% and 43.8% at 12 months after initial SRS [9, 11, 19-20]. Our favorable results may be in part due to the aggressive use of surgery, radiation therapy, and systemic therapy as indicated as part of our multidisciplinary treatment approach for these patients.

**Table 4:** Radiosurgery for Gynecological Brain Metastases in Greater than 15 Patients Abbreviations: KM = Kaplan-Meier, LC = local control, OS = overall survival, GK = Gamma Knife; DBC = distant brain control, ND = not described

Source	Number of Patients	12-month KM LC Rate	12-month KM OS Rate (from GK Treatment)	Median OS	12-month KM DBC Rate
Monaco, et al. [19]	27	ND	15%	5 months	ND
Ogino, et al. [20]	16	ND	31%	9.5 months	ND
Shepard, et al. [10]	16	ND	75% for Ovarian Ca	22.3 months for Ovarian Ca	ND
			33% for Endometrial Ca	8.3 months for Endometrial Ca	
Matsunaga, et al. [9]	70	89.90%	43.80%	8 months	ND
Shin, et al. [11]	26	ND	13%	9.5 months	ND
Our Series	33	84.30%	54.90%	15 months	65.80%

We identified primary disease histology to be a statistically significant predictor of KM OS estimates, with cervical cancer patients having a median survival of 17 months, endometrial cancer patients having a median survival of six months, and ovarian cancer patients having a median survival of 16 months (p = 0.03). This finding had not completely been elucidated by prior studies on this topic, although one prior study by Shepard, et al. illustrated a similar survival benefit seen when comparing ovarian carcinoma and endometrial carcinoma (22.3 months vs 8.3 months, respectively). No other factors in our study had a statistically significant impact on KM OS estimates. 

Limitations of our study include the lack of available cause of death data, as well as a lack of analysis on the various chemotherapeutic agents that may have been employed in the treatment of each individual patient. Studies with larger sample sizes are needed to further elucidate the significance of primary histology on brain control and OS and to further confirm our overall findings. Additionally, our study has the inherent limitations of any retrospective study.

However, due to the limited data currently available, this study importantly supports the use of Gamma Knife radiosurgery for the treatment of patients with gynecological malignancies in one of the larger cohort of patients reported to date. 

## Conclusions

In summary, this study provides retrospective outcomes from a single institution utilizing Gamma Knife radiosurgery in the treatment of brain metastases from gynecological malignancies. We were able to achieve excellent local control rates with acceptable rates of toxicity.  
